# The Impact of Early Childhood Caries on Oral Health-Related Quality of Life of Children and Caregivers Residing in Rural and Urban Areas of the Rangareddy District

**DOI:** 10.25122/jml-2020-0063

**Published:** 2020

**Authors:** Syed Nazia Tabassum, Abhinaya Reddy Tupalli, Sampath Reddy Cheruku, Mohammed Abidullah, KTSS Rajajee, Thekiya Altaf Hussain

**Affiliations:** 1. Department of Pedodontics & Preventive Dentistry, Dr. Shashi’s Dental Studio, Aramghar, Hyderabad, Telangana, India; 2. Department of Pedodontics & Preventive Dentistry, Sri Sai College of Dental Surgery, Vikarabad, Hyderabad, Telangana, India; 3. Department of Dental and Biomedical Sciences, Faculty of Dentistry, Al Baha University, Al Baha, Saudi Arabia; 4. Anil Neerukonda Institute Of Dental Sciences, Bheemili, Visakhapatnam, Andhra Pradesh, India; 5. Department of Orthodontics, SVS Institute of Dental Sciences, Mahabubnagar, Telangana, India

**Keywords:** Early childhood caries, early childhood oral health Impact scale, oral health-related quality of life, pediatric dentist

## Abstract

Early childhood caries is a condition that impacts oral health-related quality of life of children’s development and well being and also affects parents’ work hours and poses a financial burden on them.

Our objective was to study and compare the impact of early childhood caries on the quality of life of preschool children aged 22–70 months and their caregivers in an urban and rural population using the early childhood oral health impact scale.

The study was conducted on children of the Rangareddy district, Telangana state, aged between 22 –70 months affected by early childhood caries and their parents/guardians. The subjects were given a questionnaire to measure the early childhood oral health impact scale, and the filled questionnaires were analyzed and tabulated.

The mean early childhood oral health impact scale and domain scores for the rural population were significantly higher than that of the urban population signifying a more mediocre quality of life. There was a weak positive and insignificant relationship between early childhood caries and the early childhood oral health impact scale in the rural population, whereas there was a moderately strong, significant positive relationship between the two in the urban population.

Oral health-related quality of life of young children enables parents and caregivers to implement positive dental care practices.

## Introduction

Oral health contributes to general health, enabling us to properly eat, speak, and socialize with no active disease, discomfort, or embarrassment. Children differ from adult patients in that they need primary caregiver’s assistance for health care. Assessing oral health-related quality of life (OHRQoL) among adults has been studied in the past, but children’s OHRQoL has not been given attention [1, 2].

The American Academy of Pediatric Dentistry defines early childhood caries (ECC) as ‘the presence of one or more decayed, missing due to caries, or filled tooth surfaces in any primary teeth in children under 6 years of age’. Apart from impacting OHRQoL with pain and abscess formation, ECC also impairs a child’s physical development and may also reduce children’s ability to learn [3, 4].

For parents and caregivers, ECC results in a work-loss and financial loss as they have to stay at home for a caring child or take them to a pediatric dentist for treatment procedures. Hence, ECC is recognized as a public health problem [5, 6].

There is a need for a better understanding of the OHRQoL concept to make suitable clinical decisions, find out treatment needs, and execute public health programs [3, 4]. This study was carried to evaluate the relationship between OHRQoL and ECC among preschool children and their caregivers in an urban and rural population of the Rangareddy district, Telangana state.

## Material and Methods

The study was conducted on children between 22 and 70 months affected by ECC and their parents/guardians. This study was performed in the Rangareddy district, Telangana. The subjects of the rural background were randomly selected from children who are attending village Anganwadi centers. The subjects with an urban background were selected from children attending preschools.

### Exclusion Criteria:

1. Children without ECC
2. Medically compromised children, such as children suffering from systemic diseases or physical/learning disabilities and children/accompanying parents who were not willing to participate in the study.

### Questionnaire:

A questionnaire of the items to measure the Early Childhood Oral Health Impact Scale (ECOHIS) consisting of child and family impact sections was used, and parents were asked to fill them. Three questionnaires were used; the Telugu, Urdu, and English versions were available, according to the convenience of the parents. Validation of the questionnaires was performed on linguistic and socioeconomic parameters. The questionnaire containing the ECOHIS parameters included the Telugu and Urdu languages, for the benefit of individuals who do not understand English. Before the commencement of the study, informed consent was obtained from the parents and principal of the schools attended by the study participants.

## Results

The total number of subjects was 458; 238 subjects belonged to rural areas and the remaining 220 to urban areas. The mean age of rural children was 4.51 years, and that of children from urban areas was 4.30 years (Table 1). Males were predominant in rural children and females in urban children (Table 2).

**Table 1:** Mean age of rural and urban children.

**Age**	**Mean**	**Std. Deviation**	**N**
**Rural**	4.51	0.86	238
**Urban**	4.30	0.79	220

**Table 2:** Gender of rural and urban children.

**Gender**	**Female**	**Male**
**Rural**	100	138
Urban	119	101
Total	219	239

**Figure 1:**
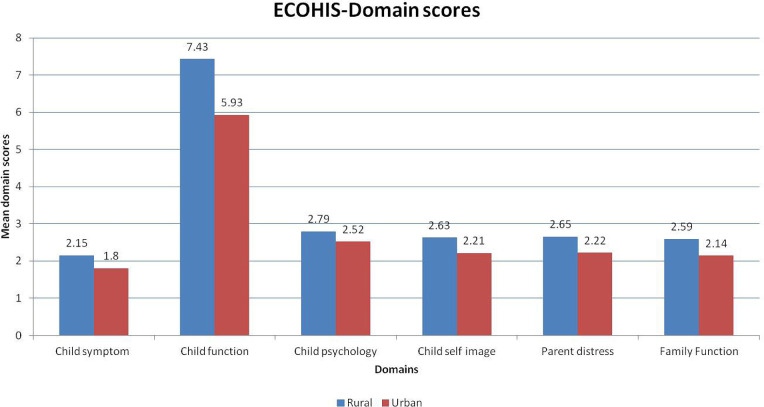
The impact of ECC on the various domains of OHRQoL.

**Figure 2:**
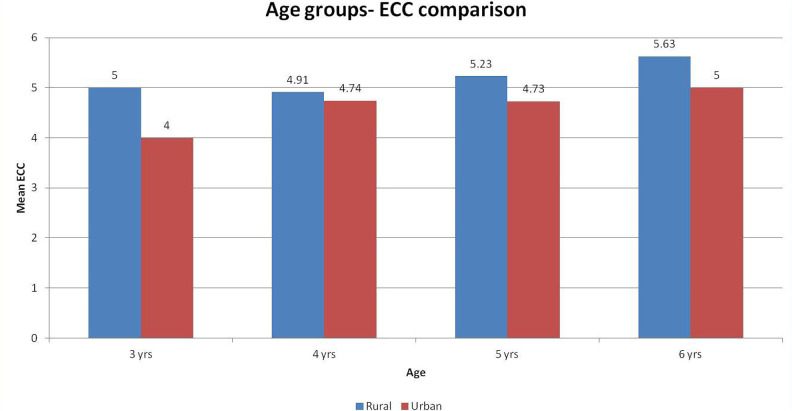
Comparison of age groups and ECC.

Unpaired t-tests were used to compare the mean ECOHIS and mean domain scores between urban and rural populations. The mean ECOHIS and domain scores for the rural population were significantly higher than that of the urban population, signifying poorer Qol (Table 3 and Figure 1). One way ANOVA test was used to compare the mean ECC amongst different age groups in rural and urban populations, respectively. There was no significant difference amongst the groups regarding the different age groups (Table 4 and Figure 2).

**Table 3:** The impact of ECC on the various domains of OHRQoL.

**Domains**	**Rural**		**Urban**		**p-value**
	**Mean**	**SD**	**Mean**	**SD**	
**Child symptom domain**	2.15	1.13	1.80	1.05	<0.001
**Child function domain**	7.43	3.66	5.93	2.86	<0.001
**Child psychological domain**	2.79	1.39	2.52	1.14	0.02
**Child Self-image domain**	2.63	1.23	2.21	0.78	<0.001
**Parent distress domain**	2.65	1.38	2.22	0.70	<0.001
**Family function domain**	2.59	1.41	2.14	0.56	<0.001
**ECOHIS**	20.29	8.92	16.81	5.74	<0.001

Note: Used test - Unpaired t - test.

**Table 4:** The mean ECC amongst different age groups in urban and rural populations.

**Group**	**Age group**	**ECC**		**N**	**p-value**
		**Mean**	**Std. Deviation**		
**Rural**	3 yrs	5.00	3.35	12	0.20(NS)
	4 yrs	4.91	2.74	105	
	5 yrs	5.23	2.53	83	
	6 yrs	5.63	2.51	38	
	**Total**	5.14	2.65	238	
**Urban**	3 yrs	4.00	3.27	22	0.94(NS)
	4 yrs	4.74	3.34	87	
	5 yrs	4.73	2.86	88	
	6 yrs	5.00	3.42	23	
	**Total**	4.69	3.14	220	

Note: Used test - One way ANOVA for rural and urban children.

The majority of rural children belonged to the upper lower class (29%), followed by the lower middle class (22.5%), whereas the majority of urban children belonged to the upper-middle class (29.9%) followed by the lower middle class (13.10%) (Table 5). Unpaired t-test and Mann Whitney U test were used to compare the mean ECC scores in males and females in rural and urban populations. There were no significant differences based on gender in either of the populations (Table 6).

**Table 5:** Distribution of samples according to socioeconomic classes.

	**Modified Kuppuswamy Scale Classification**					**Total**
**Class**	**Upper** **class**	**Upper-middle** **class**	**Lower-middle** **class**	**Upper-lower class**	**Lower** **class**	
**Rural**	0	0	103	133	2	238
	0.00%	0.00%	22.50%	29.00%	0.40%	52.00%
**Urban**	5	137	60	18	0	220
	1.10%	29.90%	13.10%	3.90%	0.00%	48.00%
**Total**	5	137	163	151	2	458
	1.10%	29.90%	35.60%	33.00%	0.40%	100.00%

**Table 6:** Comparison of ECC based on gender in the urban and rural areas.

	**Gender**	**Mean** **ECC**	**Std. Deviation**	**p-value**	**Significance**
**Rural**	**Female**	5.27	2.83	0.50	NS
	**Male**	5.04	2.52		
**Urban**	**Female**	4.62	2.93	0.74	NS
	**Male**	4.76	3.39		

Note: Used test - Rural: Unpaired t test; Urban: Mann Whitney U test.

Pearson’s correlation coefficient was used to determine the correlation between ECC and ECOHIS. There was a weak positive and insignificant relationship between ECC and ECOHIS in the rural population, whereas there was a moderately strong, significant positive relationship between ECOHIS and ECC in the urban population (Table 7).

**Table 7:** Correlation of the ECOHIS scores in rural and urban children.

**Correlations**			
	**Group**	**ECOHIS**	**ECC**
**Pearson correlation coefficient**	Rural	Correlation Coefficient	0.024 (weak)
		Sig. (2-tailed)	0.456 (NS)
	Urban	Correlation Coefficient	.533** (moderate)
		Sig. (2-tailed)	<0.001 (S)

## Discussion

It has been known that oral health status affects various aspects of QOL; in children, ECC also has an impact on the child’s wellbeing. ECOHIS evaluates the effects of oral health problems on the child and parents [2].

Many tools were developed to assess the QOL for older children and adults, but for children less than 6 years of age, very little research has been done. A comprehensive questionnaire should consider the social, economic, and cultural differences level of maturity and social, cognitive, and emotional development along with the linguistic ability of the children [2, 3].

The proforma used by us had a total of 16 items with 12 items targeting the child impact, and four concerned the family impact, based on the studies of Juniper et al. [3, 4]. The advantages of this method of questionnaire-based data collection are the low cost, bias-free, ease of study, and the possibility to investigate a large sample [5, 6].

We carried this study intending to compare ECC’s ECC’s impact on the quality of life of preschool children aged 22–70 months and their caregivers in the urban and rural population using the ECOHIS.

We found that the mean age of rural and urban children was 4.51 and 4.30 years, respectively. In the study of Genderson et al., the average age of the participants was 12.8 ± 1.2 [5]. The population targeted by Acharya and Tandon was children below 71 months of age, with a mean of 52.06 months [2]. A similar age group (a mean of 50.4 months) was selected in the study of Filstrup et al. [7].

We found that males were predominant in rural children and females in urban children. In the study by Genderson et al., 52.8% were females [5]. In most studies, most informants were mothers, as was observed in our study. It has been recognized that fathers may have limited knowledge of their children’s activities and feelings [5-7]. No significant difference was observed between fathers and mothers as informants in our study, although only a small proportion of the informants were fathers.

Our first objective was to compare the ECC in different socioeconomic strata and its effects on the QoL of children and their families using the Kuppaswamy scale. It takes into consideration the education status, occupation, and financial status of the head of the family. This scale has been revised over the years and includes different categories like upper, upper-middle, lower-middle, upper-lower, and lower socioeconomic status. In our study, we found that most of the rural children belonged to the upper lower class (29%) followed by the lower middle class (22.5%), whereas the majority of urban children belonged to the upper-middle class (29.9%) followed by the lower middle class (13.10%). There was no significant difference amongst the groups regarding the different age groups [3-5].

Usually, an increase in ECC experience has been associated with low household income [7, 8] and was seen in our study as well. However, Al-Hasani et al. [9] and Olatosi et al. [10] found that those children whose caregivers had relatively high monthly incomes experienced ECC more often than those whose caregivers had lower income. The explanation given from these two studies was that there is likely to be a higher purchasing power of cariogenic snacks and drinks for children among those caregivers with higher incomes.

Our second objective was to assess the number of carious teeth in children with ECC. We found that the mean ECC score in rural children of 3, 4, 5 and 6 years age groups in rural children was 5, 4.91, 5.23 and 5.63, respectively, with a total mean of 5.14 years. On the other side, the mean ECC score in urban children of 3, 4, 5 and 6 years age groups was 4, 4.74, 4.73 and 5, respectively, with a total mean of 4.69 years.

Our third objective was to evaluate the impact of ECC on the various domains of OHRQoL. We found that the mean ECOHIS and domain scores for the rural population were significantly higher than that of the urban population, signifying poorer QoL. The impact of ECC on the symptoms domain of the child had a mean score of 2.15 in rural children and 1.80 in urban children, the difference being statistically significant (P<0.01). The impact of ECC on the function domain of the child had a mean score of 7.43 in rural children and 5.93 in urban children, the difference being statistically significant (P<0.01).

The impact of ECC on the psychological domain of the child had a mean score of 2.79 in rural children and 2.52 in urban children, the difference being statistically insignificant (P=0.02). The impact of ECC on the child’s self-image domain had a mean score of 2.63 in rural children and 2.21 in urban children, the difference being statistically significant (P<0.01). The impact of ECC on the parent’s distress domain had a mean score of 2.65 in rural children and 2.22 in urban children, the difference being statistically significant (P<0.01). The impact of ECC on the family function domain had a mean score of 2.59 in rural children and 2.14 in urban children, the difference being statistically significant (P<0.01).The mean ECOHIS score was 20.29 in rural children and 16.81 in urban children, the difference being statistically significant (P<0.01).

In our study, the findings concerning dental caries among preschool children were consistent with those of previous studies, that is, dental caries was higher among children belonging to lower socioeconomic groups [11, 12].

Our fourth objective was to determine the effect of dental caries on the children’s OHRQoL in different age groups. We used the One way ANOVA test to compare the mean ECC among different age groups in rural and urban populations. Also, there was no significant difference amongst the groups considering the different age groups.

Our fifth objective was to compare the impact of dental caries on children’s OHRQoL between the two genders. Unpaired t-test and Mann Whitney U test were used to compare the mean ECC scores in males and females in rural and urban populations, respectively. There were no significant differences based on gender in either of the populations. The mean ECC in urban female and male children was 4.62 and 4.76, respectively, whereas the mean ECC in rural female and male children was 5.27 and 5.04, respectively.

Maro and Khabuka reported higher ECC experience among male children compared to female children [13]. According to Ngatia et al. [14] and Njoroge et al. [15], who reported similar findings among preschool children in the Kenyan population, male children have more inadequate oral hygiene practices as compared to female children.

Other studies have found a higher ECC prevalence among female children compared to male children. Folayan et al. found a prevalence of 66.7% among females compared to 33.3% among males in children below 71 months [16].

We suggest that there is a need to investigate the possible effect of gender as a risk factor in ECC development.

We also found that the mean ECOHIS and domain scores for the rural population were not significantly different based on gender. Pearson’s correlation coefficient was used to determine the correlation between ECC and ECOHIS. There was a weak positive and insignificant relationship between ECC and ECOHIS, whereas there was a moderately strong, significant positive relationship between ECOHIS and ECC in the urban population.

When analyzed in this study, the relationship between gender, age, and OHRQoL, showed that neither age nor gender had a significant association with ECOHIS in the bivariate analysis or the multivariate analysis. Our findings are similar to Li MY et al. [1]. We did not find any significant increase in the mean ECC score with the age of children. However, several other studies reported an increase in ECC experience with the age of the children, and this could be attributed to long-term exposure of the teeth to cariogenic factors [14, 15].

Further studies are needed to determine the association and correlation between the perceived impact and dental condition using indicators that allow discrimination among the different states of the dental caries process and dental treatments.

## Conclusion

OHRQoL was found to be related to ECC. OHRQoL of young children enables parents and caregivers to implement positive dental care practices. Better dental care is required to improve children’s OHRQoL in India, and this can be achieved by the coordination of the government and private dental surgeons, especially pediatric dentists.

## Conflict of Interest

The authors declare that there is no conflict of interest.
